# Strain Engineering of Anisotropic Electronic, Transport, and Photoelectric Properties in Monolayer Sn_2_Se_2_P_4_

**DOI:** 10.3390/nano15090679

**Published:** 2025-04-30

**Authors:** Haowen Xu, Yuehua Xu

**Affiliations:** School of Microelectronics and Control Engineering, Changzhou University, Changzhou 213164, China; s22060809001@smail.cczu.edu.cn

**Keywords:** two-dimensional materials, first-principles calculations, strain engineering, electronic properties, carrier transport properties, optoelectronic properties

## Abstract

In this study, we demonstrate that the Sn_2_Se_2_P_4_ monolayer exhibits intrinsic anisotropic electronic characteristics with the strain-synergistic modulation of carrier transport and optoelectronic properties, as revealed by various levels of density functional theory calculations combined with the non-equilibrium Green’s function method. The calculations reveal that *a*-axis uniaxial compression of the Sn_2_Se_2_P_4_ monolayer induces an indirect-to-direct bandgap transition (from 1.73 eV to 0.97 eV, as calculated by HSE06), reduces the hole effective mass by ≥70%, and amplifies current density by 684%. Conversely, *a*-axis uniaxial expansion (+8%) boosts ballistic transport (*a*/*b*-axis current ratio > 10^5^), rivaling black phosphorus. Notably, a striking negative differential conductance arises with the maximum *I*_peak_/*I*_valley_ in the order of 10^5^ under the 2% uniaxial compression along the *b*-axis of the Sn_2_Se_2_P_4_ monolayer. Visible-range anisotropic absorption coefficients (~10^5^ cm^−1^) are achieved, where −4% *a*-axis strain elevates the photocurrent density (6.27 μA mm^−2^ at 2.45 eV) and external quantum efficiency (39.2%) beyond many 2D materials benchmarks. Non-monotonic strain-dependent photocurrent density peaks at 2.00 eV correlate with hole effective mass reduction patterns, confirming the carrier mobility of the Sn_2_Se_2_P_4_ monolayer as the governing parameter for photogenerated charge separation. These results establish Sn_2_Se_2_P_4_ as a multifunctional material enabling strain-tailored anisotropy for logic transistors, negative differential resistors, and photovoltaic devices, while guiding future investigations on environmental stabilization and heterostructure integration toward practical applications.

## 1. Introduction

The rapid development of integrated circuits has driven the continuous demand for performance enhancement in electronic devices, positioning two-dimensional (2D) semiconductors as promising candidates for applications in field-effect transistors (FETs) and photoelectric conversion systems [1,2,3,4,5,6,7,8]. However, critical limitations persist in existing 2D materials: transition metal dichalcogenides (TMDs) like MoS_2_ and ReS_2_ suffer from restricted carrier mobility [9,10,11,12], while high-mobility phosphorenes (e.g., black phosphorus and violet phosphorus) exhibit poor environmental stability due to oxidation susceptibility [13,14,15]. Furthermore, the narrow band gap of materials such as silicene and germanene increases heat loss and reduces energy conversion efficiency [16,17]. To address these challenges, it is essential not only to explore novel 2D materials through density functional theory (DFT) computational simulations but also to develop property-modification strategies like strain engineering, doping, and van der Waals heterostructure construction [18,19].

As a particularly effective approach, strain engineering enables the precise modulation of electronic structures in 2D materials owing to their exceptional mechanical flexibility. Appropriate mechanical strain enables the precise modulation of bandgap magnitude, bandgap type, and band curvature in two-dimensional materials. This strain engineering strategy allows the bandgap range to be tuned to 0.77–1.19 eV for optimal channel material performance in electronic devices or 1.1–1.6 eV for photovoltaic applications [20,21,22]. Hence, the controlled manipulation of band structures holds promise for enhancing electronic transport and optoelectronic characteristics in functional devices. This capability has been successfully demonstrated in graphene, hexagonal boron nitride (hBN), TMDs, and MXenes, opening new avenues for designing high-performance nanodevices [23,24,25,26,27].

Recent attention has been focused on a novel family of ternary 2D materials with puckered honeycomb structures—triphosphides and triarsenides (general formula AX_3_, where A = P/As and X = group II/XIII/XIV/XV elements) [28,29,30,31]. Two-dimensional triarsenides (CaAs_3_, GeAs_3_) demonstrate exceptional carrier mobility exceeding ~3 × 10^4^ cm^2^ V^−1^ s^−1^ [28,32], while their triphosphide counterparts (GeP_3_, SnP_3_) are renowned for their high optical absorption coefficients (~10^5^ cm^−1^) across the visible-to-infrared spectrum [30,33]. To expand this material family beyond conventional stoichiometries, recent theoretical studies have proposed structural derivatives through unit cell doubling and selective chalcogen substitution, leading to the prediction of Ge_2_S_2_P_4_ and Sn_2_S_2_P_4_ monolayers with exceptional photocatalytic water-splitting capabilities [34,35].

Particularly noteworthy is the high-performance two-dimensional monolayer (ML) Sn_2_Se_2_P_4_ predicted by Trung et al., exhibiting phonon-dispersion-validated dynamic stability and ultra-high anisotropic mobility properties [36]. An ab initio molecular dynamics (AIMD) simulation was employed to heat the Sn_2_Se_2_P_4_ monolayer at 500 K for 10 ps, showing sufficient thermal stability. As determined by the Perdew–Burke–Ernzerhof (PBE) calculations, the Sn_2_Se_2_P_4_ monolayer exhibits an indirect bandgap of 1.04 eV, which decreases and transitions to a direct bandgap under biaxial compressive strain. Remarkably, this material demonstrates an outstanding carrier transport performance, with electron and hole mobilities reaching 5.4 × 10^3^ cm^2^ V^−1^ s^−1^ and an extraordinary 7.4 × 10^4^ cm^2^ V^−1^ s^−1^, respectively. These superior properties position Sn_2_Se_2_P_4_ as a promising candidate for next-generation electronic and optoelectronic devices. While existing studies have primarily investigated the effects of biaxial strain on this material, the comprehensive modulation of electrical transport and optoelectronic properties in Sn_2_Se_2_P_4_ through uniaxial strain—a more flexible and controllable tuning strategy—remain systematically underexplored. Notably, whether uniaxial strain can effectively modulate the bandgap of Sn_2_Se_2_P_4_ into the optimal range for optoelectronic devices while preserving or even enhancing its exceptional carrier mobility remains an open question. Motivated by the pivotal role of strain engineering in optimizing the performance of two-dimensional (2D) materials, we systematically investigate the electronic structure, carrier transport properties, and optoelectronics characteristics of Sn_2_Se_2_P_4_ monolayers under uniaxial strain, aiming to establish a strain-dependent framework for multifunctional device design.

In this work, we systematically investigate the strain-tunable anisotropic properties of orthorhombic Sn_2_Se_2_P_4_ monolayers through first-principles calculations. Our density functional theory (DFT) analysis reveals the following: (1) direction-dependent bandgap engineering from indirect to direct transitions under uniaxial compression along the *a*-axis; (2) strain-modulated effective mass reductions (holes under *a*-axis compression) correlated with enhanced transport properties; (3) non-equilibrium Green’s function (NEGF) calculations demonstrating anisotropic current–voltage characteristics with current ratios exceeding 10^5^ and strain-enhanced negative differential conductance (NDC) effects; and (4) optoelectronic property optimization under specific strain conditions, achieving an external quantum efficiency (39.2%) and photocurrent density (6.27 μA mm^−2^) surpassing conventional photovoltaic materials. These findings establish Sn_2_Se_2_P_4_ as a versatile platform for developing strain-engineered nanoelectronic and optoelectronic devices.

## 2. Calculational Methods

### 2.1. Details of Structural Optimization and Electronic Structure

The geometric optimization and electronic properties of the Sn_2_Se_2_P_4_ monolayer were calculated using density functional theory (DFT) implemented in the QuantumATK software (ver. S-2021.06) [37,38]. The Perdew–Burke–Ernzerhof (PBE) formulation of the generalized gradient approximation (GGA) was employed in conjunction with the linear combination of atomic orbitals (LCAO) method [39]. The PseuDodojo pseudopotential was adopted to replace the atomic all-electron potential [40], while the medium-precision numerical basis was used for the wave function expansion. A real-space density mesh cutoff of 105 Hartree was applied, and the first Brillouin zone of Sn_2_Se_2_P_4_ was sampled with a 4 × 3 × 1 k-point grid. Structural optimization convergence was achieved when the maximum force on each atom decreased below 0.001 eV Å^−1^. A vacuum spacing of 25 Å was implemented along the non-periodic direction to eliminate interlayer interactions. To enhance the accuracy of bandgap results, the Heyd–Scuseria–Ernzerhof (HSE06) hybrid functional was subsequently employed [41].

### 2.2. The Calculational Method for Carrier Transport Properties

We employed the NEGF method based on DFT to calculate the carrier transport properties [42,43]. In DFT calculations, again, the PBE functional with the LCAO method and PseuDodojo pseudopotentials were utilized for calculating the carrier transport. For current–voltage (*I*−*V*) characteristic calculations, Brillouin zone sampling was implemented with *k*-grid configurations of 1 × 3 × 132 and 4 × 1 × 76 along the *a*-axis and *b*-axis directions, respectively. The *I*−*V* characteristics of the two-probe system were subsequently derived through Landauer–Büttiker formalism [44]:

(1) $$I\left(V_{bias}\right)=\frac{2e}{h}\int T\left(E,\varepsilon_{L},\varepsilon_{R}\right)\times \left[f_{R}\left(E,\varepsilon_{R}\right)-f_{L}\left(E,\varepsilon_{L}\right)\right]dE$$

where $V_{bias}$ represents the bias voltage added to both sides of the electrodes and is defined as $eV_{bias}=\varepsilon_{R}-\varepsilon_{L}$. The Fermi energy levels of the left and right electrodes are denoted as $\varepsilon_{L}$ and $\varepsilon_{R}$, respectively. *E* represents the electrons. *T* is the carrier transport coefficient, and the Fermi Dirac distribution of the left and right electrodes are denoted as $f_{L}(E,\varepsilon_{L})$ and $f_{R}(E,\varepsilon_{R})$, respectively.

### 2.3. Calculational Method for Photocurrent

The photocurrent density (*J*_ph_) was calculated through the integration of NEGF methodology within the first-order perturbation theory framework, based on the first-born approximation [45,46]. The perturbation induced by electron–photon interactions was defined through the Hamiltonian, expressed as:

(2) $$\hat{H}={\hat{H}}_{0}+\frac{e}{m_{0}}A\cdot \hat{p}$$

where ${\hat{H}}_{0}$ is the Hamiltonian of the two-probe device, *e* denotes the electron charge, $m_{0}$ represents the free electron mass, $\hat{p}$ is the momentum operator, and *A* is defined as the electromagnetic vector potential. The transmission coefficients can be obtained using [47]:

(3) $$T_{\alpha }\left(E\right)=Tr\left\{i\Gamma_{\alpha }\left[\left(1-f_{\alpha }\right)G_{ph}^{<}+f_{\alpha }G_{ph}^{>}\right]\right\}$$

Tr denotes the trace operator. $G_{ph}^{<}$ and $G_{ph}^{>}$ are the lesser and greater Keldysh Green’s functions approximate to the first order, while $\Gamma_{\alpha }$ and $f_{\alpha }$ are the line width function and Fermi distribution for the α (left or right) electrode. *J*_ph_ is formulated as [48,49]:

(4) $$J_{ph}=\frac{e}{S\hbar }\int \frac{dE}{2\pi }\sum_{\alpha }T_{\alpha }\left(E\right)$$

Furthermore, the functional, basis, pseudopotentials, and *k*-point grids employed in the photocurrent calculations remained identical to those adopted in carrier transport simulations.

## 3. Results and Discussion

### 3.1. Structural and Electronic Properties of Sn_2_Se_2_P_4_

**Figure 1:**
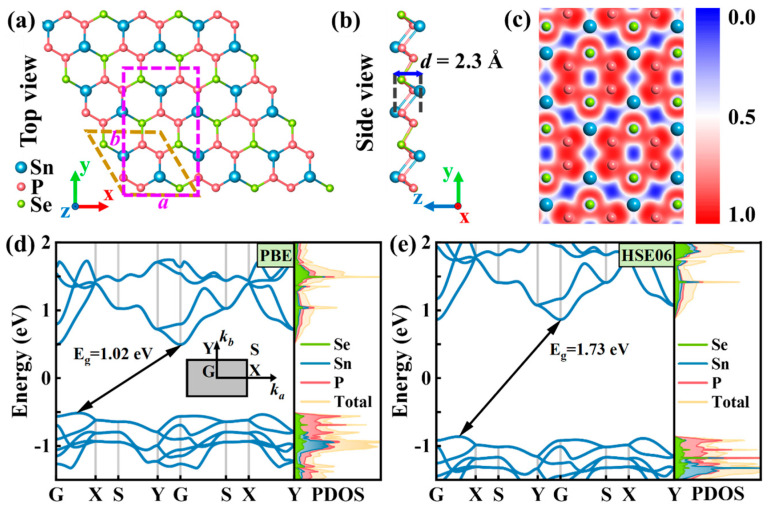
(**a**) Top and (**b**) side views of the crystal structure of the Sn_2_Se_2_P_4_ monolayer. (**c**) In-plane electron localization function map of the Sn_2_Se_2_P_4_ monolayer. Band structure and partial density of the state of the Sn_2_Se_2_P_4_ monolayer calculated at the (**d**) PBE and (**e**) HSE06 level of theory. The inset in (**d**) shows the Brillouin zone.

The Sn_2_Se_2_P_4_ monolayer was constructed by substituting two P atoms in the SnP_3_ hexagonal unit cell with Se atoms [33]. Subsequent geometric optimization using the Vienna ab initio simulation package (VASP) refined the atomic coordinates with an energy convergence threshold of 1 × 10^−6^ eV. Figure 1a,b present the top and side views of the optimized 2D Sn_2_Se_2_P_4_ monolayer, respectively. To facilitate carrier mobility calculations, the hexagonal primitive cell was transformed into an orthogonal unit cell, demarcated by bronze and purple dashed lines. The optimized monolayer exhibited a thickness of *d* = 2.30 Å and lattice constants of *a* = 7.17 Å and *b* = 12.42 Å, aligning closely with the theoretical predictions by Trung et al. Prior computational evaluations established its thermal, dynamic, and mechanical stability [36]. The electron localization function (ELF) analysis elucidated the bonding characteristics through the spatial mapping of the electron pair distribution. As depicted in Figure 1c, the electrons are localized around the P and Se atoms, thereby revealing that Se–P and P–P bonds exhibit typical covalent features, while Sn–P and Sn–Se bonds demonstrate ionic character.

The electronic band structure of the Sn_2_Se_2_P_4_ monolayer calculated using the PBE functional exhibited indirect bandgap semiconductor characteristics with a computed bandgap of 1.02 eV (Figure 1d). To address the well-known bandgap underestimation inherent in the PBE method, we employed the HSE06 hybrid functional, resulting in a significant increase in the bandgap to 1.73 eV (Figure 1e), which aligned with previously reported data [36]. Spin–orbit coupling (SOC) effects were investigated through PBE + SOC and HSE06 + SOC band structure calculations (Figure S1), revealing a neglectable difference in the band gap values. Projected density of states (PDOS) analysis (Figure 1d,e) demonstrated that the conduction band minimum (CBM) arose from the hybridized contributions of Se, Sn, and P atomic orbitals, while the valence band maximum (VBM) was predominantly governed by P orbital states.

**Figure 2:**
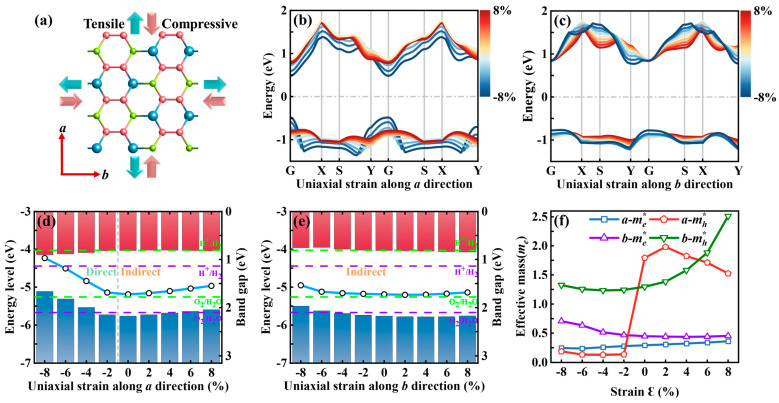
(**a**) Schematic illustration of the Sn_2_Se_2_P_4_ monolayer under uniaxial strain. The directions of tensile and compressive strains are indicated by the arrow orientations. Calculated (**b**,**c**) band structures, (**d**,**e**) band edges with band gaps, and the (**f**) effective mass of the Sn_2_Se_2_P_4_ monolayer under uniaxial strain ranging from −8% to 8% along the *a*-axis and *b*-axis, respectively.

To systematically investigate the strain-dependent modulation of electronic structures, uniaxial strains ranging from −8% to +8% were applied along the *a*- and *b*-axis of the Sn_2_Se_2_P_4_ monolayer (Figure 2a). HSE06-calculated band evolution under uniaxial strains (Figure 2b,c) revealed that the indirect bandgap characteristic persisted under b-axis strain, whereas compressive a-axis strain induced CBM-VBM degeneracy at the G-point of the Brillouin zone, triggering an indirect-to-direct bandgap transition. Concurrently, the bandgap decreased monotonically from 1.73 eV to 0.97 eV with increasing compressive strain. The strain-dependent shifts in band edge positions and HSE06-calculated band gaps along both axes are illustrated in Figure 2d,e. The redox potentials given by the Nernst equation (ESI†) for photocatalytic water splitting at pH = 0 (purple dashed) and pH = 7 (green dashed) are annotated [50], with *E*_CBM_ lying below the reduction potential and *E*_VBM_ above the oxidation potential, satisfying photocatalytic water splitting requirements. Notably, structures with *b*-axis strains of −4% to +8% optimally meet the pH = 0 criteria, while those between −4% and −8% align with pH = 7 conditions (see ESI†), demonstrating strain engineering’s precision in tailoring photocatalytic performance.

Figure 2f further reveals the strain-dependent modulation of carrier effective masses. The electron effective mass exhibits weak strain dependence along both the *a*-axis (0.25–0.36 $m_{e}^{*}$) and *b*-axis (0.46–0.71 $m_{e}^{*}$). In contrast, the hole effective masses show prominent anisotropic responses: along the *a*-axis, the hole effective mass abruptly increases to 1.98 $m_{e}^{*}$ under +2% tensile strain but sharply decreases to 0.14 $m_{e}^{*}$ at −2% compressive strain. Conversely, *b*-axis hole effective masses remain within 1.23–2.51 $m_{e}^{*}$ across all strain conditions. Notably, the significant reduction in hole effective mass (<1 $m_{e}^{*}$) within the *a*-axis compressive strain range of −2% to −8% predicts an order-of-magnitude enhancement in hole mobility, providing a theoretical foundation for optimizing carrier transport properties via strain engineering.

### 3.2. Anisotropic I–V Characteristics

**Figure 3:**
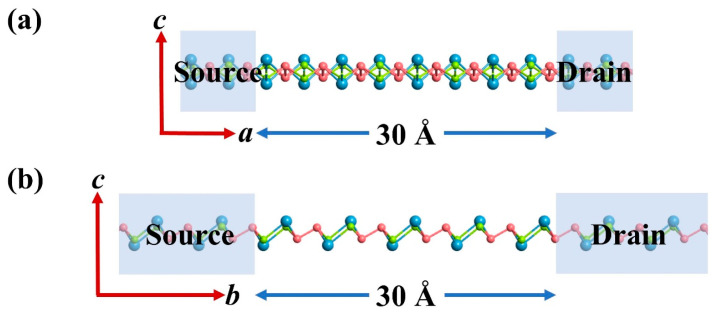
Schematic diagrams of the two-probe system for computing the transport properties of the Sn_2_Se_2_P_4_ monolayer along the (**a**) *a*-direction and (**b**) *b*-direction. Channel length was set to 30 Å.

The pronounced anisotropic carrier mobility in the Sn_2_Se_2_P_4_ monolayer—with *a*-axis hole mobility ($\mu_{h}^{a}\;$ = 7.4 × 10^4^ cm^2^ V^−1^·s^−1^, ~300 times higher than electron mobility) and b-axis electron mobility ($\mu_{e}^{b}$ = 5.4 × 10^3^ cm^2^ V^−1^·s^−1^, ~62 times greater than hole mobility)—motivated our design of the two-probe configurations to reveal the intrinsic transport behavior (Figure 3a,b). To eliminate metal–semiconductor contact barriers, homogeneous heavily doped electrodes were implemented. The hole transport channel along the *a*-axis was engineered by elevating the electrode’s VBM 0.15 eV above the Fermi level, achieving a hole density of 10^14^ cm^−2^. Conversely, the electron transport channel along the *b*-axis was configured by lowering the CBM 0.15 eV below the Fermi level, corresponding to an electron density of 10^14^ cm^−2^.

**Figure 4:**
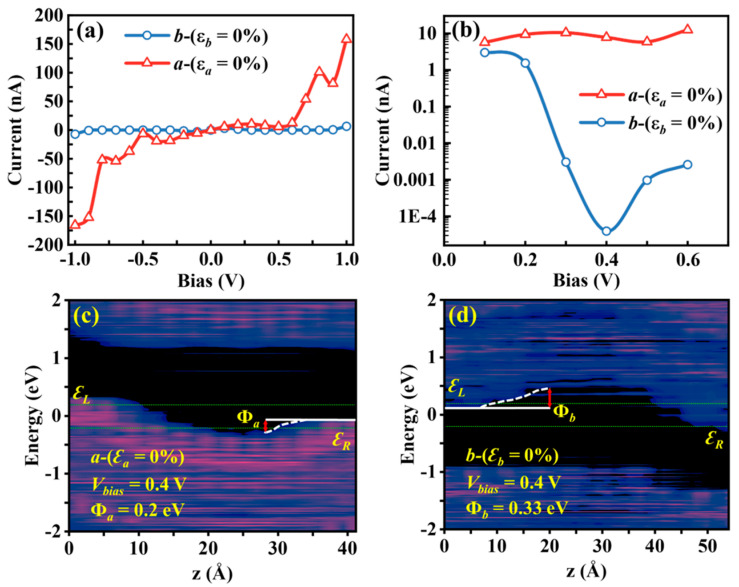
(**a**) Computed *I*−*V* characteristic curves of the unstrained Sn_2_Se_2_P_4_ monolayer. (**b**) To better observe the current changes, the logarithm scale was used to plot the *I*−*V* characteristic curve. Computed projected local density of states (PLDOS) of the unstrained Sn_2_Se_2_P_4_ monolayer at 0.4 V along (**c**) the *a*-axis and (**d**) the *b*-axis.

The unstrained-state (ε = 0%) transport properties of Sn_2_Se_2_P_4_ monolayer exhibited prominent directional dependence (Figure 4a). In the |V| ≤ 1.0 V bias range, the *a*-axis hole transport demonstrated a linear current–voltage response, achieving *I_a_* = 1.57 × 10^2^ nA at V = 1.0 V. In contrast, the *b*-axis electron transport showed abnormal negative differential conductance (NDC) near a 0.4 V bias (Figure 4b), where the current abruptly dropped to 4.0 × 10^−5^ nA, creating a current ratio of 10⁵ versus the *a*-axis transport. This phenomenon arose from the *b*-direction effective barrier height elevation (Φ*_b_* = 0.33 eV, 0.12 eV higher than the *a*-direction effective barrier height Φ*_a_*) that impeded electron transport, according to the projected local density of states (PLDOS) plots (Figure 4c,d). Consequently, focusing on this distinctive −0.6 V to 0.6 V bias window, we explored strain-modulated *I*−*V* characteristics under uniaxial strain engineering.

### 3.3. Uniaxial Strain-Modulated I–V Characteristics

#### 3.3.1. *a*-Axis Strain-Modulated *I–V* Characteristics

**Figure 5:**
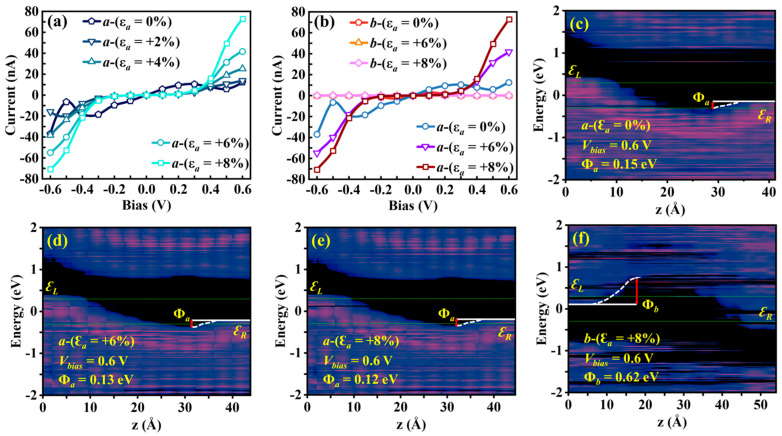
(**a**) Calculated *I*−*V* characteristic curves of the Sn_2_Se_2_P_4_ monolayer at uniaxial strains ranging from 0% to +8% in the *a*-axis. (**b**) Comparative results of *I*−*V* characteristics for *a*-axis strains (0%, +6%, +8%) calculated along the *a*-axis versus those obtained along the *b*-axis. The PLDOS of the Sn_2_Se_2_P_4_ monolayer at 0.6 V under (**c**) 0%, (**d**) +6%, and (**e**) +8% *a*-axis uniaxial strain computed along the *a*-axis; and (**f**) the +8% *a*-axis uniaxial strain calculated along the *b*-axis.

**Figure 6:**
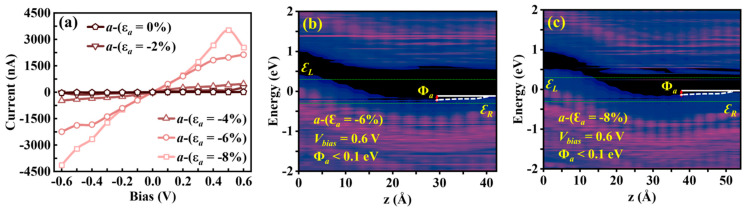
(**a**) Computed *I*−*V* characteristic curves of the Sn_2_Se_2_P_4_ monolayer under uniaxial strains ranging from −8% to 0% in the *a*-axis. The calculated PLDOS at 0.6 V under (**b**) −6% and (**c**) −8% *a*-axis uniaxial strain.

By applying uniaxial *a*-axis strain (−8% ≤ ε*_a_* ≤ +8%) to the Sn_2_Se_2_P_4_ monolayer, we observed a strong correlation between the magnitude of *a*-axis strain (tensile/compressive) and the anisotropic carrier transport properties (Figure 5 and Figure 6). Under *a*-axis tensile strain (+2% ≤ ε*_a_* ≤ +8%), the current in the low-bias regime (|V| < 0.4 V) decreased by 90% compared to the unstrained structure, while a strain-enhanced effect emerged at 0.4 V < |V| < 0.6 V: the current *I_a_* increased by 421% as ε*_a_* rose from +2% to +8% (Figure 5a). According to Figure 5c–e, PLDOS analysis revealed that tensile strain reduced the effective barrier height of the *a*-axis transport channel to Φ*_a_* = 0.12 eV at ε*_a_* = +8%, marking a 20% reduction from the unstrained value (Φ*_a_* = 0.15 eV). Notably, strain simultaneously enhanced transport anisotropy, achieving an *a*/*b*-axis current ratio of 10^5^ at V = 0.6 V for ε*_a_* = +8% (Figure 5b). This ratio matched the switching performance of black phosphorus (10^5^), fulfilling the ON/OFF ratio requirements for field-effect transistors (FETs) [51]. The phenomenon aligned with an 80% reduction in the *a*-axis effective barrier height (Φ*_a_* = 0.12 eV versus Φ*_b_* = 0.62 eV) at V = 0.6 V under ε*_a_* = +8% (Figure 5f).

In contrast, compressive *a*-axis strain (ε*_a_* = −6% and −8%) significantly enhanced the carrier transport efficiency of the Sn_2_Se_2_P_4_ monolayer (Figure 6a). At ε*_a_* = −8%, the current density *I_a_* reached 3.53 × 10^3^ nA, representing a two-order-of-magnitude enhancement over the unstrained structure. First-principles calculations revealed that this nonlinear response originated from strain-induced band reconstruction: compressive strain triggered a transition from an indirect to a direct bandgap (E_g_ decreased from 1.73 eV to 0.97 eV), accompanied by a 0.43 eV downward shift in the CBM and a 0.38 eV upward shift in the VBM toward the Fermi level (Figure 2b). PLDOS calculations further confirmed that, compared to the unstrained structure (Figure 5c), compressive strains of ε*_a_* = −6% and −8% reduced the *a*-axis effective barrier height to Φ*_a_* < 0.1 eV (Figure 6b,c), thereby markedly facilitating hole tunneling transport.

#### 3.3.2. *b*-Axis Strain-Modulated *I–V* Characteristics and NDC Effects

**Figure 7:**
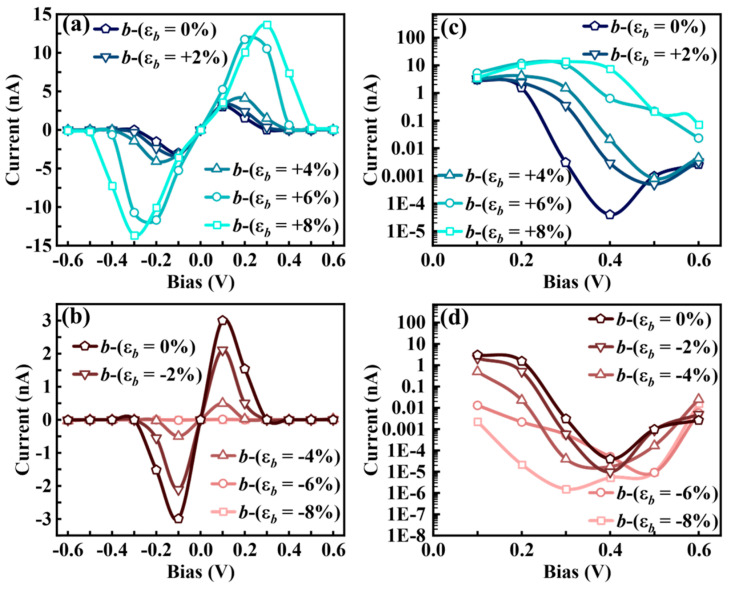
(**a**) *b*-axis 0–+8% tensile strain and (**b**) −8–0% compressive strain *I*–*V* characteristics of the Sn_2_Se_2_P_4_ monolayer, with logarithmic-scale *I*–*V* plots under (**c**) tensile and (**d**) compressive strain along the *b*-axis.

**Figure 8:**
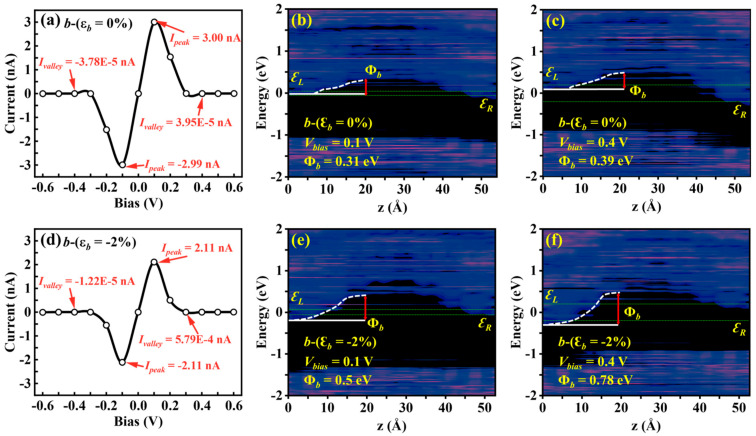
Computed (**a**) *I*–*V* characteristics and PLDOS at (**b**) 0.1 V and (**c**) 0.4 V in the *b*-axis of the unstrained Sn_2_Se_2_P_4_ monolayer along with the calculated (**d**) *I*–*V* characteristics and PLDOS at (**e**) 0.1 V and (**f**) 0.4 V for the −2% *b*-axis compressive strain counterpart.

The transport properties of the Sn_2_Se_2_P_4_ monolayer under *b*-axis strain exhibit unique NDC modulation in 0.1–0.4 V (−0.4 to −0.1 V) bias regimes. Under *b*-axis strain (ε*_b_* = 0% and −2%), the transport current initially rises within 0–0.4 V bias, abruptly decreases, and subsequently recovers beyond 0.4 V (Figure 7a,c). The calculated logarithmic-scale *I*−*V* characteristics (Figure 7b,d) reveal the pronounced NDC behavior in both the unstrained structure and ε*_b_* = −2% strained case within 0.1–0.4 V, achieving a peak-to-valley current ratio (*I*_peak_/*I*_valley_) in the order of 10^5^ (Figure 8a,d). This ratio surpasses the reported NDC effects in graphene (50–200) [52], phosphorene (25) [53], phosphorene/ReS_2_ heterostructures (4.2–6.9) [15], and MoS_2_/WSe_2_ heterostructures (10^3^) [54]. PLDOS analysis demonstrates that the effective barrier height Φ*_b_* at *V* = 0.4 V (Figure 8c,f) is significantly higher than at *V* = 0.1 V (Figure 8b,e) for both cases, leading to suppressed carrier transport. This disparity in carrier transport capacity between the peak and valley states directly induces the pronounced NDC effect.

### 3.4. Photocurrent Transport Properties

**Figure 9:**
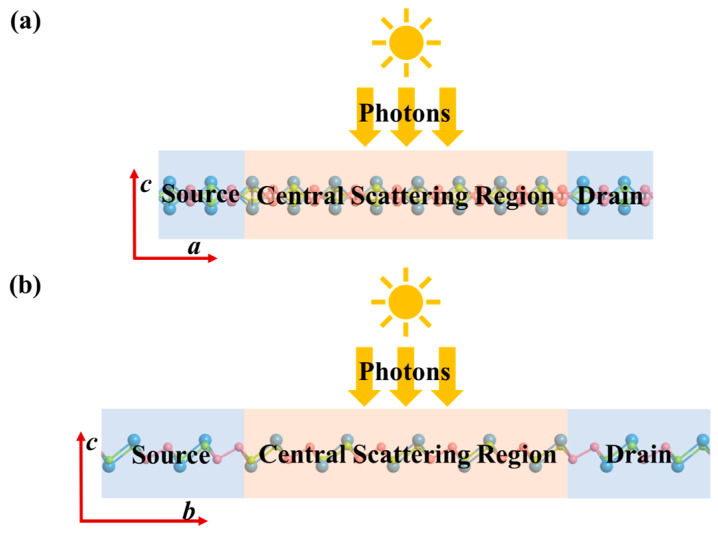
Schematic diagrams of the Sn_2_Se_2_P_4_ monolayer-based optical nanodevices along the (**a**) *a*-axis and (**b**) *b*-axis, respectively.

To investigate the optoelectronic performance of the Sn_2_Se_2_P_4_ monolayer in practical device environments, we construct optical nanodevices along the *a*- and *b*-axes, leveraging its intrinsic anisotropy. As shown in Figure 9a,b, the device features an intrinsic Sn_2_Se_2_P_4_ scattering region (length set as 50 Å) for linearly polarized light irradiation, connected to heavily doped p-type (source) and n-type (drain) Sn_2_Se_2_P_4_ semi-infinite electrodes. While the PBE functional inherently underestimates band gaps, high-concentration doping strategies align the effective band gaps with theoretical predictions, ensuring computational reliability. Building on our prior discovery that *a*-axis compressive strain (−8% ≤ ε*_a_* ≤ −2%) induces indirect-to-direct bandgap transitions, we further investigate its modulation effects on optoelectronic performance.

**Figure 10:**
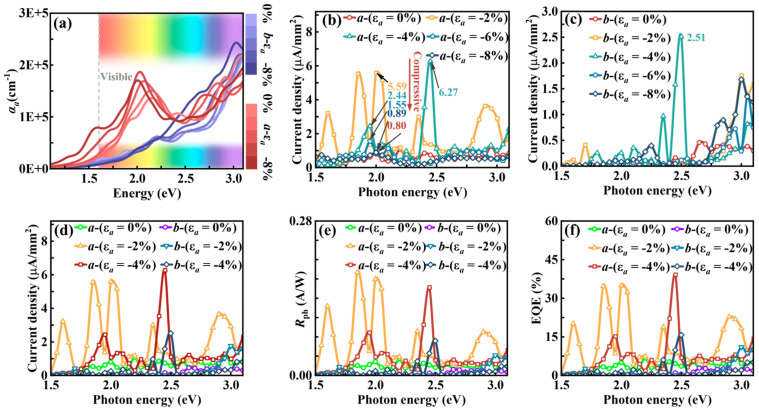
(**a**) Calculated absorption coefficients of Sn_2_Se_2_P_4_ monolayer under *a*-axis compressive strain (−8% ≤ ε*_a_* ≤ −2%) along *a*-axis (blue curves) and *b*-axis (red curves), respectively. Computed *J*_ph_ under *a*-axis compressive strain (−8% ≤ ε*_a_* ≤ −2%) along (**b**) *a*-axis and (**c**) *b*-axis, respectively. Comparative results of (**d**) *J*_ph_, (**e**) *R*_ph_, and (**f**) EQE under 0%, −2%, and −4% *a*-axis compressive strain computed along both axes.

As shown in Figure 10a, the optical absorption coefficient of the Sn_2_Se_2_P_4_ monolayer exhibits pronounced anisotropy across the visible spectrum (1.61–3.11 eV). The unstrained structure (ε*_a_* = 0%) achieves an *a*-axis absorption coefficient of ~10^5^ cm^−1^, comparable to that of the same family of ternary 2D monolayers, including Sn_2_Te_2_P(As)_4_ (10^4^–10^5^ cm^−1^) [55], Ge_2_S_2_P(As)_4_ (about 10^5^ cm^−1^) [34], SnGeS_2_As_4_ (10^5^–10^6^ cm^−1^) [56], and Sn_2_S_2_P_4_ (about 10^5^ cm^−1^) [35]. Under *a*-axis compressive strain (−8% ≤ ε*_a_* ≤ −2%), absorption is further enhanced, accompanied by distinct spectral separation between the *a*-axis and *b*-axis absorption profiles, indicating strain-induced anisotropy enhancement. This phenomenon aligns with strain-modulated carrier transport properties, confirming the synergistic control of optoelectronic and transport properties.

Photocurrent density (*J*_ph_), as a critical metric for optoelectronic device performance, quantifies the photogenerated current per unit area under illumination, reflecting the material’s light absorption and conversion efficiency. By computing the *J*_ph_, we demonstrate that strain engineering enhances the photovoltaic conversion efficiency of the Sn_2_Se_2_P_4_ monolayer. At ε*_a_* = −4%, the *a*-axis device achieves a *J*_ph_ peak of 6.27 μA mm^−2^ at 2.45 eV (Figure 10b), representing a 684% enhancement over the unstrained state (0.80 μA mm^−2^) and surpassing the InSe (0.018 μA mm^−2^) and NaCuTe (1.68 μA mm^−2^) counterparts [57,58]. In contrast, the *b*-axis device under the same strain conditions only yields 2.51 μA mm^−2^ (Figure 10c), highlighting the *a*-axis as the preferred photoelectric channel. Notably, the *J*_ph_ peak around 2.00 eV initially rises sharply from 0.80 μA mm^−2^ (ε*_a_* = 0%) to 5.59 μA mm^−2^ (ε*_a_* = −2%), then declines to 0.89 μA mm^−2^ (ε*_a_* = −8%). This trend aligns with strain-dependent hole effective mass variations (Figure 2f), suggesting carrier transport limitations under extreme strains. Overall, the ε*_a_* = −2% and ε*_a_* = −4% strains significantly enhance *J*_ph_ while preserving the anisotropy of photocurrent density.

Furthermore, photoresponsivity (*R*_ph_) and external quantum efficiency (EQE) also serve as critical metrics for evaluating optoelectronic device performance. Their expressions are defined as follows [59]:

(5) $$R_{ph}=\frac{J_{ph}}{I_{\omega }E}$$

(6) $$EQE=R_{ph}\frac{hc}{e\lambda }$$

where *E* is the photon energy, *I*_ω_ represents the photon flux, *h* denotes Planck’s constant, *c* is the speed of light, *e* is the electron charge, and *λ* represents wavelength.

We investigate the *R*_ph_ and EQE of the *a*-axis and *b*-axis devices in the Sn_2_Se_2_P_4_ monolayer, applying the same methodology as used for *J*_ph_ (Figures S2 and S3). Given the deterministic influence of photocurrent density on *R*_ph_ and EQE, their strain-dependent trends under *a*-axis compressive strain align with the photocurrent density behavior. Specifically, an optimization threshold exists within the strain modulation window: *R*_ph_ and EQE peak at ε*_a_* = −2% and ε*_a_* = −4%, with performance gains diminishing outside this range. Thus, our analysis focuses on devices under ε*_a_* = −2% and ε*_a_* = −4%.

As shown in Figure S2a, the calculated *R*_ph_ reaches 0.16 A/W at 2.45 eV (ε*_a_* = −4%) and 0.17 A/W at 2.00 eV (ε*_a_* = −2%), surpassing the reported values for MoS_2_ (0.016 A/W) and graphene (5 × 10^−4^ A/W) [60]. Similarly, Figure S3a shows EQE values of 39.2% at 2.45 eV (ε*_a_* = −4%) and 34.9% at 2.00 eV (ε*_a_* = −2%), exceeding those of SnS (22.01%) [61], NaCuTe (34.3%) [58], NaCuSe (8%) [58], and KAgSe (17.9%) [62]. Consistent with the anisotropy of photocurrent density, the *R*_ph_ and EQE retain pronounced anisotropy (Figure 10e,f), with significant enhancements over the unstrained counterpart.

In this part, we demonstrate that the Sn_2_Se_2_P_4_ monolayer under moderate *a*-axis compressive strains (ε*_a_* = −2% and ε*_a_* = −4%) simultaneously achieves enhanced light absorption, tunable optoelectronic properties, and optimized carrier transport. Its integrated performance surpasses benchmark 2D optoelectronic materials (e.g., MoS_2_, SnS) and establishes a novel material platform for designing spectrally selective and directionally sensitive integrated optoelectronic devices.

## 4. Conclusions

This study systematically unveils the anisotropic electronic, transport, and optoelectronic properties of Sn_2_Se_2_P_4_ monolayers under strain engineering through DFT and NEGF methodologies. The pristine Sn_2_Se_2_P_4_ monolayer exhibits an indirect bandgap of 1.73 eV (HSE06). Under −8% *a*-axis compressive strain, the bandgap monotonically decreases to 0.97 eV with concurrent indirect-to-direct transition and drastic hole effective mass reduction ($m_{h}^{*}$: 1.79→0.13 $m_{e}^{*}$), thereby surmounting the intrinsic mobility limitations caused by the high effective mass in conventional TMDs. Notably, ballistic transport simulations (30 Å channel length) of the Sn_2_Se_2_P_4_ monolayer reveal an *a*/*b*-axis current ratio reaching ~10^5^ at 0.4 V under ±1 V bias, comparable to black phosphorus, with sustained performance under 6–8% *a*-axis tensile strain. *b*-axis compression (0–2%) induces pronounced NDC effects (*I*_peak_/*I*_valley_ ≈ 10^5^). *a*-axis strain engineering for the Sn_2_Se_2_P_4_ monolayer induces strong anisotropy in optoelectronic responses, including optical absorption coefficients (>10^5^ cm^−1^), photocurrent density (*J*_ph_), responsivity (*R*_ph_), and external quantum efficiency (EQE). At −4% *a*-axis strain under 2.45 eV photon energy, *J*_ph_ peak reaches 6.27 μA mm^−2^ (684% enhancement versus unstrained state) with an EQE of 39.2%, significantly surpassing benchmark materials like NaCuTe (1.68 μA mm^−2^, 39.4%). Most compellingly, the non-monotonic *J*_ph_ peak evolution at 2.00 eV under a-axis compression (−8%→0%) correlates strongly with strain-dependent $m_{h}^{*}$ variations, demonstrating carrier mobility as the dominant factor in photocarrier extraction for Sn_2_Se_2_P_4_ monolayers. These findings demonstrate that the Sn_2_Se_2_P_4_ monolayer achieves the synergistic modulation of electronic–optoelectronic properties through uniaxial strain engineering, with its tailorable anisotropy establishing an ideal platform for developing multifunctional nano-devices in the post-Moore era. Future investigations should prioritize environmental stability enhancement and 2D heterostructure integration to expedite experimental validation and device implementation.

## Data Availability

The data presented in this study are available on request from the corresponding author.

## Supplementary Materials

The following supporting information can be downloaded at: https://www.mdpi.com/article/10.3390/nano15090679/s1, Figure S1: The calculated electronic band structure of the Sn_2_Se_2_P_4_ monolayer with spin–orbital coupling (SOC) (orange dash curves), and without SOC (blue curves), extracted from (a) the PBE method and (b) HSE method; Figure S2: The calculated *R*_ph_ of the Sn_2_Se_2_P_4_ monolayer along (a) the *a*-axis and (b) *b*-axis under different compressive strains; Figure S3: The calculated EQE of the Sn_2_Se_2_P_4_ monolayer along (a) the *a*-axis and (b) *b*-axis under different compressive strains. Ref. [50] is cited in the Supplementary Materials.
