# Supplementary material for: Strain Engineering of Anisotropic Electronic, Transport, and Photoelectric Properties in Monolayer Sn2Se2P4
Source: Nanomaterials (Basel). 2025 Apr 30;15(9):679. doi: 10.3390/nano15090679 (PMC12073495; doi:10.3390/nano15090679)
Supplement: Supplementary file 1 [file nanomaterials-15-00679-s001.zip › nanomaterials-3575445-supplementary.pdf]

Supplementary Materials

Strain Engineering of Anisotropic Electronic, Transport, and Photoelectric Properties in Monolayer $Sn_2Se_2P_4$

Haowen Xu[1], Yuehua Xu[1,*]

[1] School of Microelectronics and Control Engineering, Changzhou University, Changzhou 213164, Jiangsu, China

[*] Corresponding authors

Corresponding author: yhxu@cczu.edu.cn

Redox potentials calculated according to the Nernst equation:

The redox capability of photocatalytic water-splitting materials can be assessed by comparing the relative positions of the CBM, VBM, and the redox potentials of water. Therefore, as stated in the Calculational Methods section, our calculations and subsequent analyses are based on the electronic structure obtained from the HSE06 functional. An effective catalyst should function across a broad range of pH values. According to the Nernst equation[1]:

$$E_{\mathrm{H^+/H_2}} = -4.44 + \mathrm{pH} \times 0.059(\mathrm{eV}) \qquad \text{(S1)}$$

$$E_{\mathrm{O_2/H_2O}} = -5.67 + \mathrm{pH} \times 0.059(\mathrm{eV}) \qquad \text{(S2)}$$

Therefore, the redox potentials at pH = 7 are −4.03 eV for H^+/H_2 and −5.26 eV for O_2/H_2O, while at pH = 0 they are -4.44 eV for H^+/H_2 and −5.67 eV for O_2/H_2O.

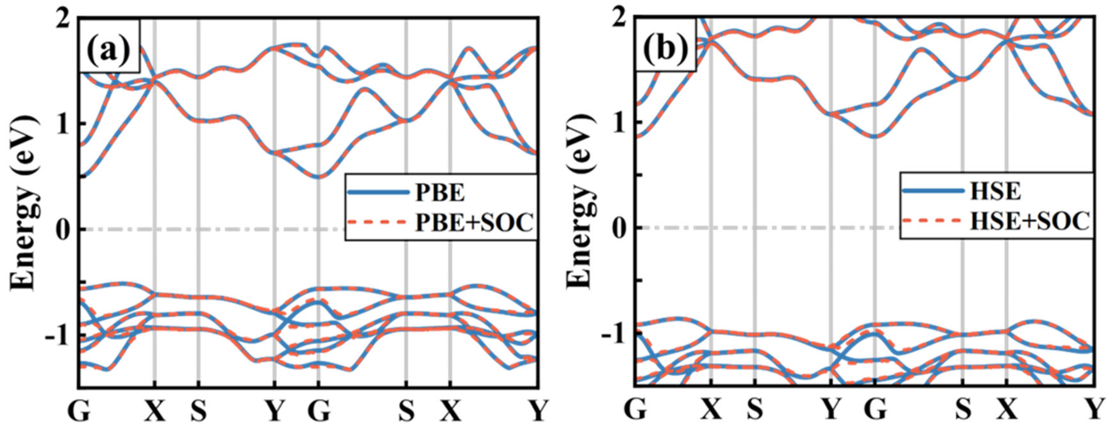

Figure S1 The calculated electronic band structure of the $Sn_2Se_2P_4$ monolayer with spin–orbital coupling (SOC) (orange dash curves), and without SOC (blue curves), extracted from (**a**) the PBE method and (**b**) HSE method.

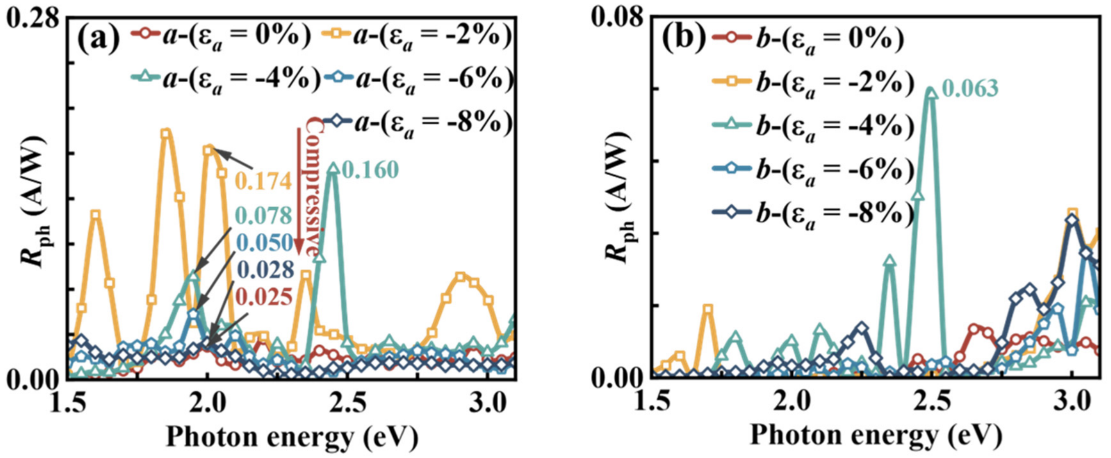

Figure S2 The calculated R_{ph} of the $Sn_2Se_2P_4$ monolayer along (**a**) the *a*-axis and (**b**) *b*-axis under different compressive strains.

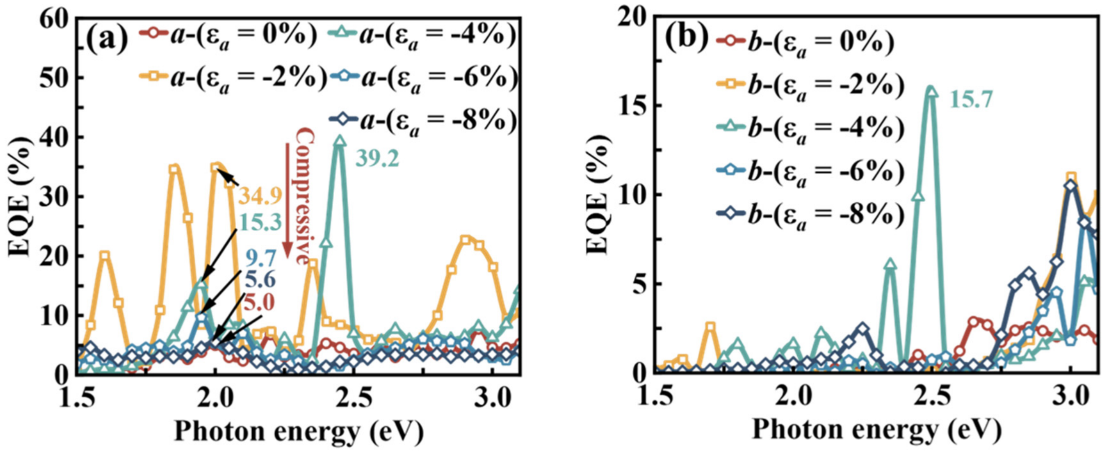

Figure S3 The calculated EQE of the $Sn_2Se_2P_4$ monolayer along (**a**) the *a*-axis and (**b**) *b*-axis under different compressive strains.

References:

1. Walczak, M.M.; Dryer, D.A.; Jacobson, D.D.; Foss, M.G.; Flynn, N.T. pH Dependent Redox Couple: An Illustration of the Nernst Equation. Journal of Chemical Education 1997, 74, 1195, doi:10.1021/ed074p1195.
